# Cutaneous Aβ-Non-nociceptive, but Not C-Nociceptive, Dorsal Root Ganglion Neurons Exhibit Spontaneous Activity in the Streptozotocin Rat Model of Painful Diabetic Neuropathy *in vivo*

**DOI:** 10.3389/fnins.2020.00530

**Published:** 2020-05-25

**Authors:** Laiche Djouhri, Asad Zeidan, Seham A. Abd El-Aleem, Trevor Smith

**Affiliations:** ^1^Department of Basic Medical Sciences, College of Medicine, QU Health, Qatar University, Doha, Qatar; ^2^Department of Histology and Cell Biology, University of Manchester, Manchester, United Kingdom; ^3^Department of Pathology, Faculty of Medicine, Minia University, Minya, Egypt; ^4^Medical Physics and Biomedical Engineering, University College London, London, United Kingdom

**Keywords:** action potential, diabetic neuropathy, nociception, primary sensory neurons, K^+^ channels

## Abstract

Diabetic peripheral neuropathic pain (DPNP) is the most devastating complication of diabetes mellitus. Unfortunately, successful therapy for DPNP remains a challenge because its pathogenesis is still elusive. However, DPNP is believed to be due partly to abnormal hyperexcitability of dorsal root ganglion (DRG) neurons, but the relative contributions of specific functional subtypes remain largely unknown. Here, using the strepotozotocin (STZ) rat model of DPNP induced by a STZ injection (60 mg/kg, i.p), and intracellular recordings of action potentials (APs) from DRG neurons in anesthetized rats, we examined electrophysiological changes in C-and Aβ-nociceptive and Aβ-low threshold mechanoreceptive (LTM) neurons that may contribute to DPNP. Compared with control, we found in STZ-rats with established pain hypersensitivity (5 weeks post-STZ) several significant changes including: (a) A 23% increase in the incidence of spontaneous activity (SA) in Aβ-LTMs (but not C-mechanosensitive nociceptors) that may cause dysesthesias/paresthesia suffered by DPNP patients, (b) membrane hyperpolarization and a ∼85% reduction in SA rate in Aβ-LTMs by K_v_7 channel activation with retigabine (6 mg/kg, i.v.) suggesting that K_v_7/M channels may be involved in mechanisms of SA generation in Aβ-LTMs, (c) decreases in AP duration and in duration and amplitude of afterhyperpolarization (AHP) in C-and/or Aβ-nociceptors. These faster AP and AHP kinetics may lead to repetitive firing and an increase in afferent input to the CNS and thereby contribute to DPNP development, and (d) a decrease in the electrical thresholds of Aβ-nociceptors that may contribute to their sensitization, and thus to the resulting hypersensitivity associated with DPNP.

## Introduction

Diabetic peripheral neuropathic pain (DPNP) is the most debilitating complication of diabetes mellitus with up to 50% of people with diabetic neuropathy (DN) having some degree of PNP (e.g., Lee-Kubli and Calcutt, 2014). Patients with DPNP usually experience a range of unpleasant positive symptoms such as spontaneous/ongoing pain, mechanical and cold hypersensitivity and dysesthesias/paresthesias, as well as negative symptoms notably heat hypoalgesia (e.g., Schreiber et al., 2015). Successful therapy for DPNP remains a challenge because currently available drugs have either limited efficacy and/or adverse side effects. Despite its high prevalence and clinical importance, the pathophysiology of DPNP is still illusive. However, several mechanisms have been implicated in its pathogenesis including mechanisms that involve structural changes in the peripheral nervous system (Polydefkis et al., 2004; Shun et al., 2004), and functional changes such as abnormal hyperexcitability of dorsal root ganglion (DRG) and CNS neurons (see (Baron et al., 2010)). Like other models of PNP, the hypersensitivity and/or ongoing pain associated with DPNP are likely to result from changes in excitability of the primary afferent neurons (Burchiel et al., 1985; Hong et al., 2004; Jagodic et al., 2007; Serra et al., 2012). Several animal studies using rodent models of DPNP have shown that both C- and A-fiber DRG neurons exhibit aberrant spontaneous activity (SA), the key characteristic of neuronal hyperexcitability (Burchiel et al., 1985; Ahlgren et al., 1992; Chen and Levine, 2001; Khan et al., 2002). While these studies suggest that both A-and C-fiber afferent fibers are involved in DPNP pathogenesis, the relative contributions of specific functional afferent subtypes that are likely to mediate different aspects of DPNP remain unknown. Indeed, little is known about the sensory receptive properties (sensory modality) of the afferent neurons involved in development and/or maintenance DPNP pathogenesis.

The primary aim of this *in vivo* study was to examine in the streptozotocin (STZ) rat model of DPNP electrophysiological changes in physiologically identified C- and A-fiber DRG neurons that might contribute to their hyperexcitability, and thereby to DPNP development. We found (5 weeks post-STZ) several significant changes in both nociceptive and non-nociceptive DRG neurons in STZ rats. These include SA in A/β-LTMs (but not in C-mechanosensitive nociceptors) that may cause dysesthesias/paresthesias experienced by many patients with DPNP. Interestingly, we also found that activation of Kv7 channels with retigabine caused membrane hyperpolarization and marked suppression of the SA in Aα/β-LTMs suggesting that these channels may be involved in mechanisms of SA generation in this subtype of Aβ-LTMs. It should be note that a decrease in expression or a change in activation properties of Kv7 channels that underlie the M current that normally acts as a “dynamic brake” on repetitive action potential discharges (see Brown and Passmore, 2009) could result in membrane depolarization, and render neurons more prone to action potential firing.

## Methods

### The Animal Model of DPNP and *in vivo* Preparation

Male Sprague–Dawley rats (250–300 g) were used in the present study. They were housed in groups of 4 per cage, in a room (with room temperature maintained between 21 and 24°C) in the animal house of Liverpool University (UK), under standard laboratory conditions with 12-h light\dark cycles. The experimental protocols were approved by the University of Liverpool Ethical review committee, and complied throughout with the UK Animals (Scientific Procedures) Act 1986. The terminal surgery and recordings were conducted under deep anesthesia (sodium pentobarbitone 60 mg/kg i.p.). At the end of experiments, animals were humanely killed with an overdose of sodium pentobarbitone. We used the STZ rat model of DPNP that involves a single injection of STZ (60 mg/kg, i.p.) after an overnight fast to reduce competition between glucose and STZ for uptake into pancreatic β-cells as reported previously (see Djouhri et al., 2019). It is noteworthy that hypoinsulinemia and hyperglycemia caused by STZ is due to its cytotoxicity to pancreatic β cells. The STZ model (model of type 1 diabetes) is more commonly used than other models of DPNP because of its low cost, rapid induction, greater stability and relative lack of toxicity to other organs (Skovso, 2014). More imoprtantly, the STZ model is known to exhibit long lasting behavioral signs of DPNP including mechanical and heat hypersensitivity (see Djouhri et al., 2019 and section “Discussion”).

### Pain Behavioral Testing

Pain behavioral testing was conducted on 10 STZ rats by an experimenter blind to the various treatment animal groups as reported recently (Djouhri et al., 2019). Briefly, paw withdrawal threshold (PWT), paw withdrawal latency (PWL), and cold escape/nocifensive behavior, were assessed on the left hind paw of each STZ rat. Mechanical hypersensitivity was indicated by a significant decrease in the mean PWT, whereas heat hypersensitivity was indicated by a significant decrease in the mean PWL to a noxious heat. Cold hypersensitivity was assessed using a hot/cold plate analgesiometer (Ugo Basile, Milan, Italy) and a stop watch to record the duration (in seconds) of the escape/nocifensive behavior (licking, lifting, guarding, shaking or biting of the hind paw, or jumping) in a 2 min period in rats before and after STZ treatment. Cold hypersensitivity was indicated by a significant decrease in the duration of cold-evoked responses as reported previously (e.g., Orio et al., 2009). STZ rats were also assessed for the presence of spontaneous foot lifting (SFL), a behavioral sign of spontaneous/ongoing pain as described previously (Djouhri et al., 2019).

### *In vivo* Electrophysiological Recordings

Intracellular recordings were made in deeply anesthetized STZ rats (*n* = 28) 5 weeks post-STZ or in control (vehicle treated) rats (*n* = 18 rats) of similar age/weight. General anesthesia was induced by sodium pentobarbitone (60 mg/kg. i.p.). The relatively large number of rats used is due to the low percentage (about 23%) of neurons with SA (see section “Results”). This means finding a spontaneously firing neuron requires 4 or more successful experiments with at least 4 neurons in each experiment. Full details of the animal preparation were as reported previously (Djouhri et al., 2018). Briefly, to allow artificial ventilation and monitoring of end-tidal CO_2_, a tracheotomy was performed. CO_2_ was maintained between 3 and 4% by adjusting the volume and rate of the respiratory pump. The left jugular vein and carotid artery were tabulated to enable, respectively, regular injections of additional doses of anesthetic (sodium pentobaribtone 10mg/kg) and monitoring of blood pressure (normally ∼80–100 mmHg). A laminectomy was performed from vertebrae L2-L6 as reported previously (Djouhri et al., 2018). Intracellular voltage recordings from somata of L4 or L5 DRG neurons were obtained using sharp glass microelectrodes filled with 3M or 1M KCl. Somatic action potentials (APs) were antidromically evoked by dorsal root stimulation with single rectangular pulses (0.03 ms duration for A-fiber neurons or 0.3 ms for C-fiber neurons). The electrical threshold (the minimum stimulus (in volts) applied to the dorsal root that evoked a somatic AP) was measured. The stimulus intensity (up to 25 V) was adjusted to twice threshold for A-fiber neurons and between 1 and 1.5 times threshold for C-fiber neurons. As reported previously (Djouhri et al., 2006), spontaneous firing/activity (SA) which is the ongoing firing in the absence of any known stimulus, was recoded (when present) before modality testing to avoid the search stimuli influencing such firing. In all neurons, the assessment period for SA was initially 2 min. The recording time was extended up to 5 min for neurons exhibiting SA during this initial observation period to calculate the rate of SA. SA rate was calculated by counting the number of action potentials during the recording period.

### Conduction Velocity, Action Potential Recordings, and Selection Criteria

The conduction velocity (CV, m/s) of each neuron was calculated by dividing the conduction distance between the recording and stimulating sites by the latency to onset of the evoked AP. Neurons were classified according to their dorsal root CVs as C (≤ 0.8m/s), Aδ (1.5–6.5 m/s) or Aα/β (>6.5 m/s) (see Fang et al., 2002). These borderlines were relatively low, for reasons discussed previously including inclusion of utilization time (Djouhri and Lawson, 2001). As reported previously (e.g., Fang et al., 2002; Djouhri et al., 2018), APs were recorded online with a CED (Cambridge Electronics Design) 1401 plus interface and the spike II programs from CED and were subsequently analyzed offline using CED Spike II program. For each neuron, various variables were measured as described previously (see (Djouhri et al., 2012)). These include CV, resting membrane potential (Em), durations of AP at base, rise time (RT) and fall time (FT) as well as AP height and AP overshoot. AHP duration to 80% recovery (AHP80%) and AHP amplitude (between Em and maximum AHP depth). were also measured. Neurons were included in the analysis only if their Ems were equal to, or more negative, than −40 mV, and an AHP.

### Sensory Receptive Properties

The sensory receptive properties of DRG neurons were identified using natural innocuous and noxious mechanical and thermal stimuli as described previously (Fang et al., 2005; Djouhri et al., 2018). Briefly, non-nociceptive neurons including low threshold mechanorecptive (LTMs) were those units that responded to non-noxious mechanical stimuli including light brushing of limb fur with a fine paint-brush, skin contact and light pressure with blunt objects. Only cutaneous Aβ-LTMs were included in the current study; they were classed as slowly adapting units or rapidly adapting units (see Fang et al., 2005; Djouhri et al., 2018). Slowly conducting C-, Aδ-fiber LTMs were not encountered in the present study. Nociceptive neurons were those that failed to respond to the non-noxious mechanical stimuli but responded either to noxious mechanical stimuli (applied with a needle, fine forceps or coarse toothed forceps) only, or to both noxious mechanical and noxious heat stimuli (hot water at 50–65°C applied with a syringe). These are referred to as C-mechano-sensitive nociceptors. C-mechano-insensitive nociceptors were not included in the present study.

### Statistical Tests

**FIGURE 1:**
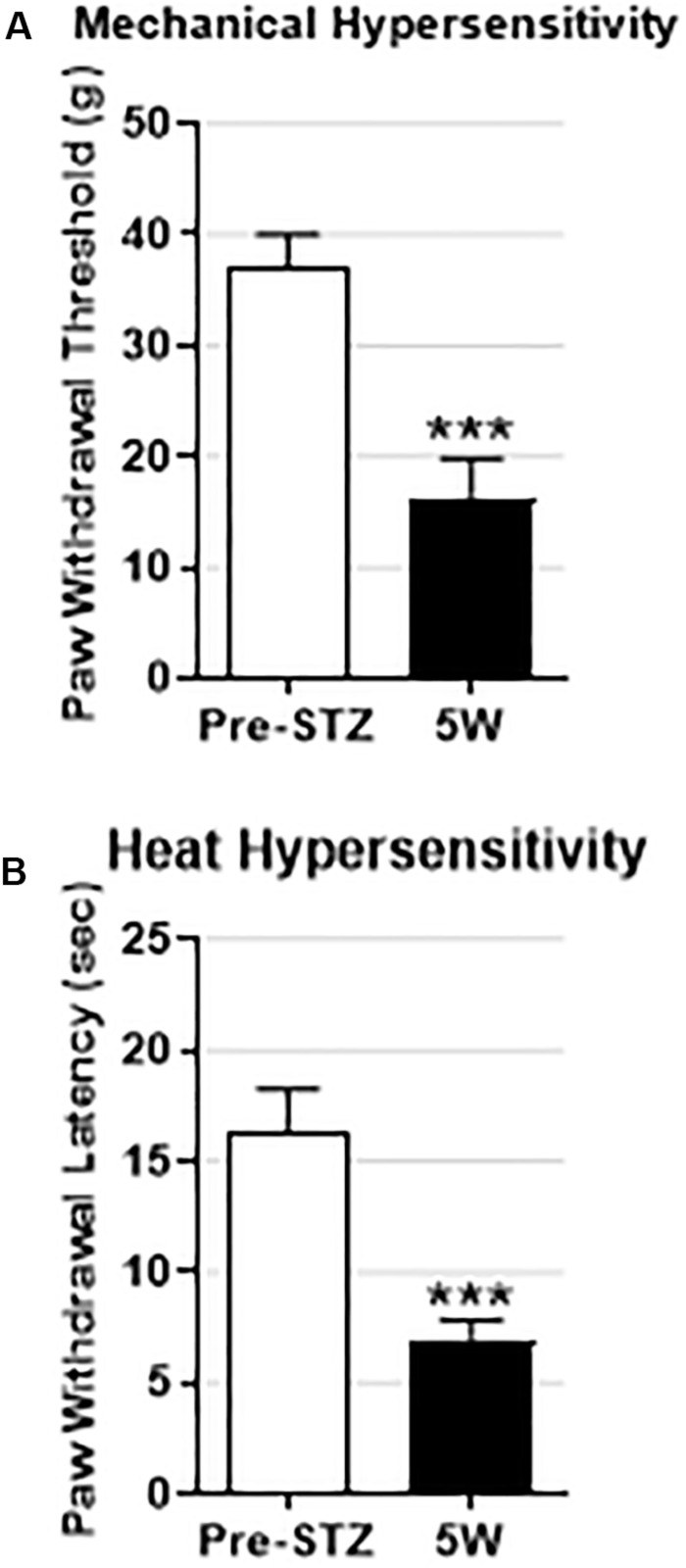
Behavioral signs of mechanical and heat hypersensitivity in STZ-diabetic rats. Data are presented as mean ± SEM. STZ treatment significantly decreased the mean paw withdrawal threshold (PWT) **(A)** and the mean paw withdrawal latency (PWL) **(B)** in STZ rats (*n* = 10) 5 weeks post-STZ. The decrease in both PWT and PWL was highly significant (*P* < 0.001) indicating that the STZ-treated rats developed mechanical and heat hypersensitivity. Comparisons are between values before STZ treatment (pre-STZ, *open bars*) and 5 weeks post-STZ (5W, *black bars*). Statistical tests were made with paired *t*-test. The level of statistical significance is: ^∗∗∗^*P* < 0.001.

**FIGURE 2:**
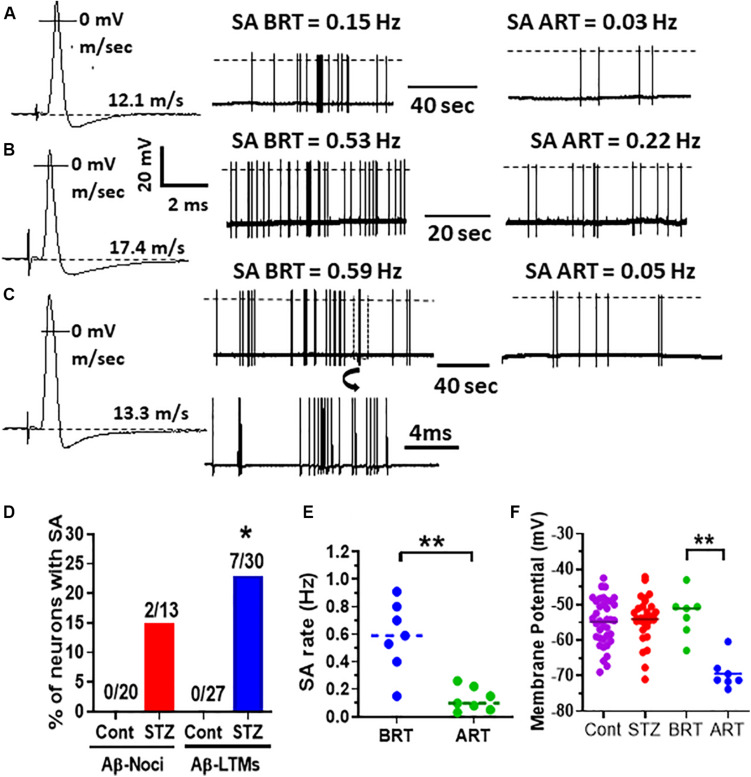
Effects of retigabine on spontaneous activity (SA) and membrane potential (Em) in Aβ-LTMs in STZ rats. Shown in panels **(A–C)** are example records of three typical spontaneously active Aβ-LTMs recorded from STZ rats before and 15 min after systemic administration of retigabine (6 mg/kg, i.v.). The left traces **(A,B** and **C,B)** are somatic APs evoked by dorsal root electrical stimulation and recorded intracellularly from these three Aβ-LTMs; the CV (m/s) of each neuron is given above the Ems shown by dotted lines. The middle traces in panels **(A–C)** are original records of these neurons firing spontaneously at different rates before retigabine administration (BRT). The traces on the right in panels **(A–C)** illustrate that 15 min after retigabine (ART), the SA rate of these neurons was reduced from 0.15 to 0.03 Hz, 0.53 to 0.22 Hz, and 0.59 to 0.05 Hz, respectively. Shown below the middle trace in panel **(C)** is part of the trace shown in panel **(C)** (in a box) on an expanded time scale (note different time scales). Panel **(D)** shows that the percentage of Aβ-LTMs with SA (but not Aβ-nociceptors) is significantly greater (Fisher exact test, *P* < 0.05) than that of control Aβ-LTMs. Panel **(E)** shows that the median SA rate (0.59 Hz) for the whole sample of Aβ-LTMs tested (*n* = 7) was significantly (*P* < 0.01) reduced to 0.09 Hz 15 min after retigabine (ART) administration. Panel **(F)** shows that retigabine (6 mg/kg, i.v.) caused a highly significant decrease (hyperpolarization) in the median resting membrane potential (Em) of the Aβ-LTMs in STZ rats (*P* < 0.001, paired *t*-test). Note that there was no significant difference in the median Em between STZ and control Aβ-LTMs. The voltage and time scales to the right of AP shown in panel **(B)** are for all three evoked APs. The dotted lines on the traces indicate AP overshoot. *P < 0.05; **P < 0.01.

**FIGURE 3:**
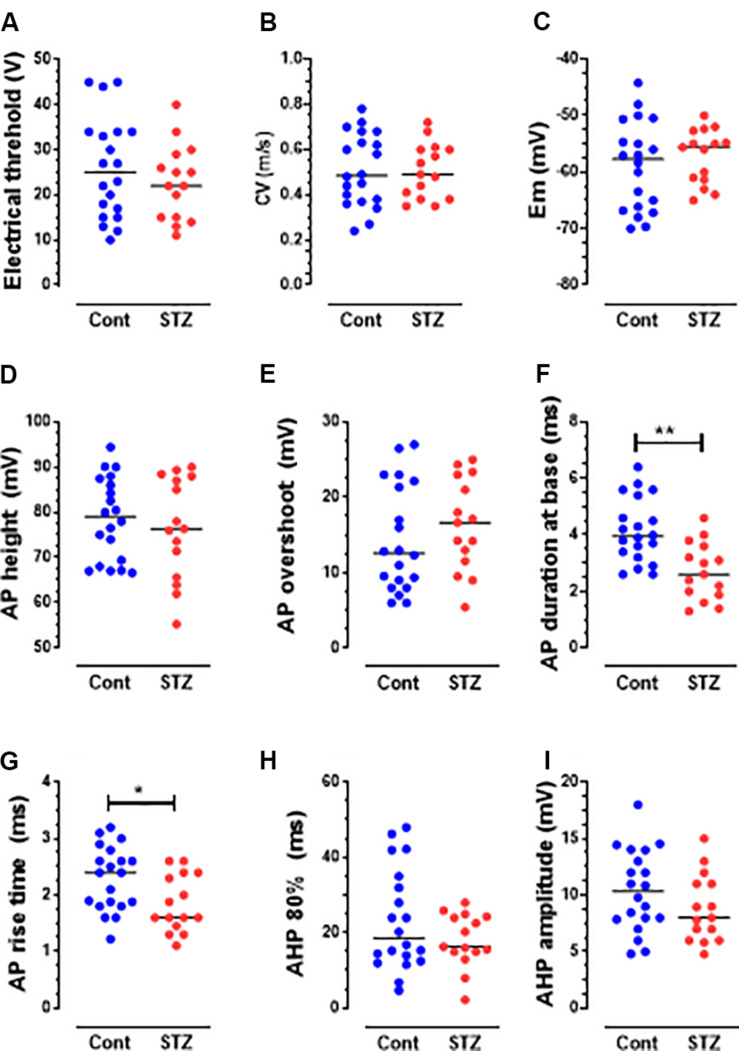
Changes in AP variables in C-fiber mechano-sensitive nociceptors in STZ rats. The graphs **(A–I)** show scatterplots of variables measured 5 weeks post-STZ. These variables are: electrical threshold **(A)**, CV **(B)**, Em **(C)**, AP height **(D)**, AP overshoot **(E)**, AP duration at base **(F)**, Rise time (RT) **(G)**, AHP duration (AHP 80%, **H**) and AHP amplitude **(I)**. Each dot in these graphs and in those shown in Figure 4 represents a value from one neuron. Medians are superimposed. Note the significant decreases in the durations of AP **(F)** and RT **(G)**, but not in other variables. Comparisons between medians in columns 1 (control, blue dots) and column 2 (STZ, red dots) were with the Mann–Whitney *U* test. The level of statistical significance is as follows: ^∗^*P* < 0.05; ^∗∗^*P* < 0.01.

**FIGURE 4:**
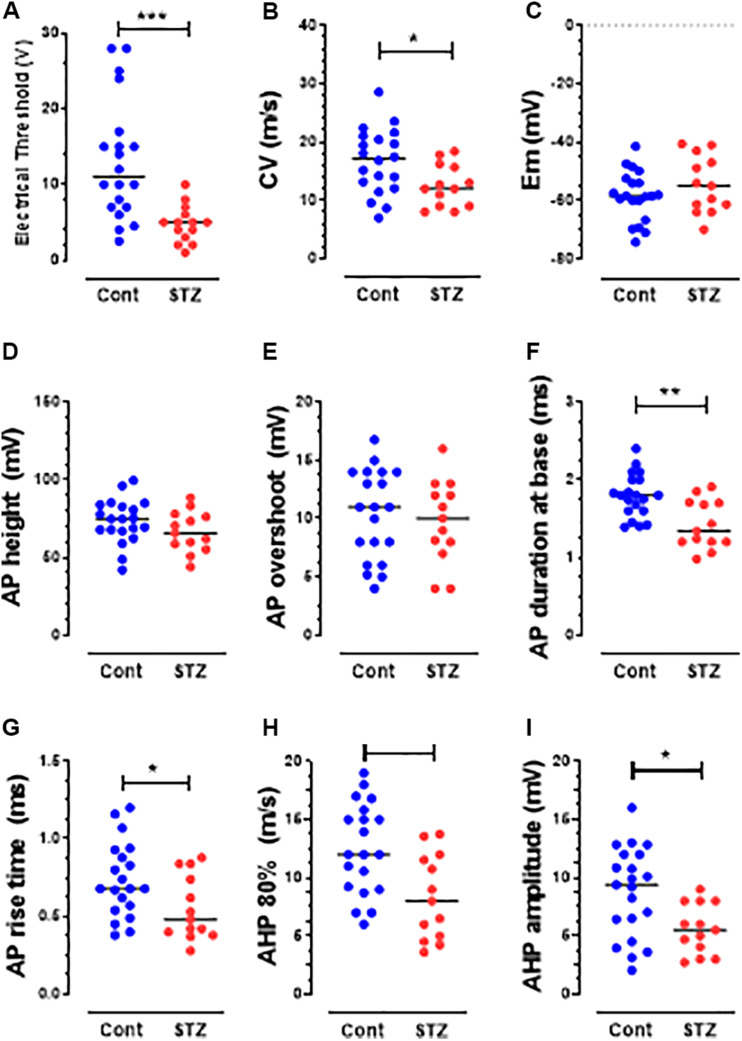
Changes in AP variables in Aβ-nociceptors in STZ rats. The variables shown are the same as those shown in Figure 3. Note the significant decreases in the CV **(B)**, durations of AP **(F)**, RT **(G)**, and AHP **(H)** and in AHP amplitude **(I)**. Details are as in Figure 2. Note that there was no significant change in the other variables: electrical threshold **(A)**, resting membrane potential **(C)**, AP height **(D)**, AP overshoot **(E)** and afterhyperpolarization duration to 80% recovery **(H)**.

The behavioral data are normally distributed (Shapiro–Wilk normality test) and are therefore presented as the mean ± SEM. Comparisons between values before and after STZ treatment were made with paired *t*-tests (Figure 1). Comparisons of percentage change were made using the Fisher exact test (Figure 2). Most of the electrophysiological data (not normally distributed) are presented as medians and compared with the nonparametric Mann–Whitney *U* test (Figures 3, 4). All tests were made using GraphPad Prism software, version 8 (GraphPad, San Diego, CA, United States), and were two-tailed. Values of *P* < 0.05 were considered significant. Statistical significance: ^∗^*P* < 0.05, ^∗∗^*P* < 0.01, and ^∗∗∗^*P* < 0.001.

## Results

### STZ Rats Exhibit Behavioral Sings of Mechanical and Heat, but Not Cold, Hypersensitivity or Spontaneous Pain

We have previously shown that STZ-diabetic rats exhibit behavioral signs of mechanical and heat hypersensitivity, but not cold hypersensitivity or spontaneous/ongoing pain (Djouhri et al., 2019). To confirm that the STZ-rats on which the present electrophysiological experiments were conducted also show behavioral indices of DPNP, we assessed pain behaviors in 10 rats including 7 STZ-rats from which the spontaneously firing Aβ-LTMs were recorded (see below). Consistent with our previous study (Djouhri et al., 2019), we found 5 weeks post-STZ that all STZ-rats exhibited: (1) significant decreases in the mean PWT and PWL (*P* < 0.01) indicating that they developed behavioral signs of mechanical and heat hypersensitivity respectively (Figure 1), and (2) no behavioral signs of cold hypersensitivity or spontaneous/ongoing pain (data not shown).

### *In vivo* Intracellular Voltage Recordings in Control and STZ Rats

*In vivo* intracellular voltage recordings were made from 125 physiologically identified DRG neurons that met the acceptance criteria defined in the methods. Only C-mechano-sensitive nociceptors, Aβ-nociceptors and cutaneous Aβ-LTMs were included in the present study. Other subtypes were either not included (e.g., Aα/β-muscle spindle afferent neurons) or not encountered including C-LTMs, C-cooling, Aδ-nociceptors and Aδ-LTMs. Of the 125 neurons recorded, 67 neurons (20 C-nociceptors, 20 Aβ-nociceptors, and 27 cutaneous Aβ-LTMs) were recorded from L4/L5 DRGs in control rats, and 58 neurons (15 C-nociceptors, 13 Aβ-nociceptors, and 30 cutaneous Aβ-LTMs) were recorded from STZ rats.

### STZ Induces Spontaneous Firing in Aβ-Nociceptors and Aβ-LTMs but Not C-Mechanosensitive Nociceptors

None of control C-nociceptors (0/20), Aβ-nociceptors (0/20) or cutaneous Aβ-LTMs (0/27) exhibited spontaneous activity (SA). Of the STZ neurons, none of the C-nociceptors (0/15) fired spontaneously, but 23% (7/30) and 15% (2/13) of cutaneous Aβ-LTMs and Aβ-nociceptors showed SA, respectively (Figure 2). Figure 2 also shows example records of three typical Aβ-LTMs with SA. These neurons fired spontaneously at a rate of 0.15 Hz (Figure 2A), 0.53 Hz (Figure 2B), and 0.59 Hz (Figure 2C). As shown in Figure 2D the incidence of Aβ-LTMs, but not Aβ-nociceptors with SA is significant (*P* < 0.05 Fisher exact test). The median SA rate for the whole sample of Aβ-LTMs was 0.59 Hz (Figure 2E).

### Activation of K_v_7 Channels With Retigabine Suppressed STZ-Induced SA in Aβ-LTMs

We have recently shown that retigabine given intraperitoneally at a dose of 15mg/kg attenuated mechanical hypersensitivity in STZ rats (Djouhri et al., 2019). Given that PNP is due partly to SA in DRG neurons (see section “Introduction”), we sought to determine whether activation of K_v_7 channels with retigabine modulates SA in Aβ-LTMs that we see in STZ-rats. Interestingly, we found that intravenous administration of retigabine at a dose of 6 mg/kg substantially reduced SA rate in all Aβ-LTMs examined (*n* = 7) as illustrated for the three Aβ-LTMs shown in Figures 2A–C. Indeed, 15 min after retigabine injection, the rate of SA in the neuron shown in Figure 2A was reduced by ∼80% (from 0.15 to 0.03 Hz), whereas in the other two neurons, the SA rate was reduced by 58% (from 0.53 to 0.22, Figure 2B) and 91% (from 0.59 to 0.05 Hz Figure 2C). The median SA rate for the whole sample of Aβ-LTMs tested was significantly (*P* < 0.01) reduced by ∼85% (from 0.59 to 0.09 Hz) 15 min after retigabine administration (Figure 2E). Only one of the two spontaneously firing Aβ-nociceptors was examined for the effects of retigabine; its SA rate was also reduced (by ∼70%) 15 min after by retigabine injection (not shown). The other Aβ-nociceptive neuron was lost before testing.

### Retigabine Causes Membrane Hyperpolarization in Spontaneously Active Aβ-LTMs

We compared resting membrane potential (Em) in STZ Aβ-LTMs with those of control Aβ-LTMs because EM could influence SA. As shown in Figure 2F, there was no significant difference in the median Ems of Aβ-LTMs between STZ and control rats. However, the Ems of Aβ-LTMs were significantly hyperpolarized 15 min after retigabine administration (*P* < 0.001, paired *t*-test). The median Em decreased from −51 to −71 mV (Figure 2F). Retigabine had no significant effects on other AP variables in Aβ-LTMs (Supplementary Figure S1).

### STZ Induces Decreased Electrical Thresholds in Aβ-but Not C-Nociceptors

To avoid the risk of sensitizing and/or losing the recorded DRG neurons, we did not measure their mechanical thresholds (see section “Discussion”). However, we compared the dorsal root electrical thresholds of control and STZ C-and Aβ-nociceptors, and found that the median electrical threshold of Aβ-nociceptors (Figure 4A) (but not C-nociceptors, Figure 3A) was significantly (*P* < 0.05) lower than that of control Aβ-nociceptors.

### STZ Induces Other Changes in Electrophysiological Properties of C-and Aβ-Nociceptors and Aβ-LTMs

Several changes in electrophysiological variables were found in both C-and Aβ-nociceptors in STZ-rats. These include significant decreases in: (1) AP and rise time (RT) durations in C-nociceptors (Figures 3F,G) and Aβ-nociceptors (Figures 4F,G) and (2) AHP duration and amplitude in Aβ- nociceptors (Figures 4H,I). The decreased AP duration in both C-and Aβ-nociceptors was largely due to a significant decrease in the duration of RT (Figures 3G, 4G) but not in fall time (data not shown). There was no significant changes in the other variables measured including CV, Em, AP fall time, AP amplitude, AP overshoot and electrical threshold exhibited in C-nociceptors (Figure 3). However, there was a highly significant (*P* < 0.001) decrease (by 80%) in the median electrical threshold in Aβfiber nociceptors (Figure 4A). Furthermore, as shown in Figure 4 there was significant decreases in the CV, AP duration, RT AHP duration and AHP amplitude in Aβ-nociceptors. We also examined whether the aforementioned variables also change in subtypes of cutaneous Aα/β-LTMs. The significant changes found in cutaneous Aβ-LTMs (grouped together) were decreased CV, AHP duration and AHP amplitude (Supplementary Figure S1). The faster AP and AHP kinetics and decreased AHP amplitude induced by STZ in A-fiber nociceptive and LTMs are likely to cause repetitive firing in these neurons resulting in increased afferent input to the CNS.

## Discussion

We report several novel electrophysiological changes in Aβ-LTMs, and C-and Aβ-nociceptors in STZ-rats that are likely to contribute to DPNP pathogenesis. These include: (1) increased incidence of SA in Aβ-LTMs that may result in dysesthesias/paresthesias associated with DPNP, and (2) faster AP and AHP kinetics, and reduced electrical thresholds (C-and/or Aβ-nociceptors) that may lead to their repetitive firing and sensitization respectively, and thereby contribute to development of DPNP. Furthermore, we found that activation of K_v_7 channels with retigabine suppressed STZ-induced SA, and caused membrane hyperpolarization in Aβ-LTMs, suggesting that the reduction in SA rate was a direct consequence of the hyperpolarizing effects of retigabine.

We used the STZ model, a well-established model of DPNP, that has been shown by numerous studies to exhibit long lasting behavioral signs of DPNP including mechanical and heat hypersensitivity (see Djouhri et al., 2019 and references therein). Consistent with those studies and with our recent study (Djouhri et al., 2019) we found that STZ-diabetic rats developed mechanical and heat hypersensitivity 5 weeks post-STZ. However, unlike other models of PNP such as those induced by traumatic nerve injury or chemotherapy that exhibit spontaneous/ongoing pain behavior and cold hypersensitivity (Djouhri et al., 2006, 2012; Al-Mazidi et al., 2018), we found that STZ-diabetic rats do not show these behaviors, in agreement with our recent study (Djouhri et al., 2019). It is noteworthy, however, that STZ model validity as a model of DPNP was challenged by a number of investigators (Romanovsky et al., 2004; Cunha et al., 2009; Bishnoi et al., 2011).

One of the interesting and novel findings of the present study is that Aβ-LTMs, but not C-mechanosensitive nociceptors, exibited signficant SA in STZ rats. Serra and his co-workers (Serra et al., 2012) also found, using microneurographic recordings from patients with diabetic neuropathy and STZ rats, significant SA in C-mechanoinsensitive, but not C-mechanosensitive nociceptors, but they did not record from afferent A-fibers. Other studies that investigated the impact of diabetes on excitability of primary afferent neurons/fibers have been inconsistent. Indeed, some studies reported that SA occurs almost exclusively in C-fibers in diabetic BB/Wistar rats (Burchiel et al., 1985), while other studies found no SA in this subtype of afferent fibers in STZ rats (Ahlgren et al., 1992; Chen and Levine, 2001). A significant increase in incidence of C-fibers with SA but not in Aδ-or Aβ-afferents was also reported by another study (Khan et al., 2002). However, these co-workers found that all CV groups in STZ-rats exhibited a significant increase in SA frequency and lower activation thresholds and augmented responses to mechanical stimuli. In contrast, another study (Chen and Levine, 2001) found no significant change in mechanical thresholds in STZ-diabetic rats. The causes for the apparent discrepancy between these various studies are not clear, but might be due to differences in the nerve type used for recordings, rat strains, time interval post-STZ and/or different recording methodologies.

Another interesting finding of the present study is that activation of K_v_7 channels with retigabine caused membrane hyperpolarization and profound suppression of SA in Aβ-LTMs. These findings suggest that retigabine exerts its anti-allodynic effects in STZ rats (Djouhri et al., 2019) by causing membrane hyperpolarization in this subpopulation of DRG neurons. Retigabine has also been shown to induce membrane hyperpolarization in several neuronal types, including DRG (Passmore et al., 2003) and spinal cord (Rivera-Arconada and Lopez-Garcia, 2005) neurons, as well as axotomized afferent neurons in mice (Roza and Lopez-Garcia, 2008). The hyperpolarizing effect of retiagbine is likely to be due to a shift in the K^+^ channel opening to more negative potentials (Rundfeldt and Netzer, 2000; Tatulian et al., 2001; Schwarz et al., 2006; Vervaeke et al., 2006). Although previous studies have implicated Aβ-afferent neurons (Xu et al., 2015), but not capsaicin-sensitive C-nociceptors (Khan et al., 2002) in STZ-induced mechanical hypersensitivity in mice, our current findings extend those findings and show that the functional identity of those Aβ-afferent neurons, at least in the rat, is Aβ-LTMs and probably not Aβ-nociceptors.

Retigabine has also been shown to block or reduce: (1) SA induced by saphenous nerve injury in almost all C-fibers and two putative Aβ-LTMs tested (Bernal et al., 2016), (2) SA in small dissociated DRG neurons following spinal cord injury (Wu et al., 2017), and in axotomized sensory fibers (Roza and Lopez-Garcia, 2008), (3) excitability of C- fibers in humans suffering from polyneuropathy (Lang et al., 2008), and (4) C- and Aδ-fiber discharges induced by heat stimuli (see (Brown and Passmore, 2009)). It is known that retigabine works by activating Kv7 channels mediating the low threshold and non-inactivating outward K^+^ (M) current that results from homo- or heteromeric assemblies of KCNQ proteins (Brown and Adams, 1980; Jentsch, 2000). The exact site of retigabine action remains to be determined, but it could be any of the anatomical compartments of DRG neuron because: (a) Kv7 channels are expressed in the somata of DRG neurons, and in their peripheral and central terminals (see (Du et al., 2018)), and (b) retigabine effects have been described at the nodal membrane of myelinated axons in peripheral nerve (Devaux et al., 2004; Schwarz et al., 2006) as well as at the somata level and at peripheral and central terminals of primary afferent neurons (see (Brown and Passmore, 2009).

It is noteworthy that retigabine exhibits non-Kv7 channel effects including positive allosteric modulation of GABA subtypes as well as blockade of voltage-gated Ca^2+^ and Kv2.1 channels (see Stas et al., 2016 and refrences therein). The Kv7 channel inhibitor XE991 also has been shown to have off-target effect on Na^+^ channels (Sun et al., 2019). Therefore, the possibility that changes in neuronal excitability caused by these Kv7 channels modulators are due to their non-Kv7 channel effects cannot be excluded. To ensure that the observed effects of retigabine on SA are mediated by K_v_7 channels, ideally one should conduct further experiments using a K_v_7 inhibitor such as XE991 to show that the effects of retigabine on SA in Aβ-LTMs are prevented by pre- or co-treatment with a K_v_7 channel blocker. However, since XE991 has been found to induce repetitive AP firing in rat lumbar DRG neurons (Barkai et al., 2017), administration of XE991 before or with retigabine is likely to induce SA in non-spontaneously firing DRG neurons. This would make interpreting the findings of such experiments very difficult. Nevertheless, further experiments are required to determine whether the other electrophysiological changes in Aβ-nociceptors and Aβ-LTMs including changes in AP and AHP variables are mediated by KV7 channels. However, the findings of such experiments should be interpreted with caution because, as noted above, both retigabine and XE991, the most widely used modulators of KV7 channels, have off-target effects. Indeed, XE991 may not be an ideal compound to evaluate the influence of blocking channels on nerve excitability, as suggested previously (Sun et al., 2019).

Our current findings of changes in AP and AHP variables in Aβ-nociceptors or Aβ-LTMs are in agreement with those of a previous *in vitro* study (Kriz and Padjen, 2003) that also reported other changes in Aβ-fibers from isolated sural nerves in STZ-rats. These *in vitro and in vivo* preclinical findings are consistent with the clinical findings of impaired perception of vibration (see Garrow and Boulton, 2006) which is known to be mediated by Aβ-fibers (e.g., Claus et al., 1993). Our results of decreased CV in Aβ-nociceptors in STZ rats which is likely to result for demyelination, are also in agreement with those of other animal studies (e.g., Chen and Levine, 2001) and with clinical studies that showed slowing of CV in A-fibers in patients with DN (Herrmann et al., 2002). In contract, another clinical study reported a reduction in CV of C mechano-sensitive fibers, but not C-mechano-insensitive fibers in patients with DN (Orstavik et al., 2006). The findings that Aβ-LTMs exhibit SA in STZ-rats may result in unpleasant sensations (dysesthesias/paresthesias) experienced by many patients with DPNP. Furthermore, SA in primary afferent neurons is believed to trigger and/or maintain central sensitization (see e.g., Baron et al., 2010), promoting hypersensitivity to normally innocuous stimulus (allodynia). In the presence of central sensitization during PNP conditions, nerve impulses carried centrally from the periphery along Aβ-LTMs are believed to be amplified and acquire access to the central nociceptive system, and thereby contribute to mechanical hypersensitivity associated with PNP. Normally central sensitization is driven by inputs from C-nociceptors (see Latremoliere and Woolf, 2009; von Hehn et al., 2012), but during chronic pain states, Aβ-LTMs are thought to exhibit phenotypic changes that would render them capable of triggering or maintaining central sensitization (Liu et al., 2000). On the other hand, spontaneous firing in Aβ-LTMs is expected, under normal conditions, to reduce transmission of pain signals at the spinal cord level via the gate control mechanism (Melzack and Wall, 1965). However, it is possible that this inhibitory mechanism is overridden by facilitatory spinal mechanisms involved in chronic pain conditions.

One of the major strengths of this study is that it is the first *in vivo* study that investigated electrophysiological changes in different subtypes of physiologically identified DRG neurons. We believe this is important because, as noted above, subtypes of DRG neuron are likely to contribute to different aspects of DPNP. We also believe that it is important to investigate these DRG neurons *in vivo* in their native environment that changes during chronic pain states in a complex way that cannot be readily mimicked *in vitro* (see Djouhri et al., 2018). Indeed, loss *in vitro* of the chemical environment including target-derived trophic factors such as NGF and BDNF, and dissociation-induced damage of DRG neurons profoundly alters properties of these neurons including their normal electrophysiological values against which comparisons are made (Kerekes et al., 2000). One limitation of the present study is that we did not routinely measure the mechanical thresholds of neurons, but precise determination of thresholds requires application of more stimuli to tissues that increases the risk of losing the recording. Another potential limitation of our study is the use of the general anesthetic pentobarbital that my affect the excitability of DRG neurons via modulating some ion channels. However, changes in the excitability of DRG neurons that we see in STZ rats are unlikely to be due to effects of pentobarbital because it was used in both control and STZ rats and because pentobarbital has been shown to have negligible effects on excitability of cutaneous Aβ-mechanoreceptive afferents (Cheng et al., 2013).

In conclusions, we have shown, in an established rat model of DPNP, that Aβ-LTMs, but not C-mechanosensitive nociceptors, exhibit significant SA, and that activation of K_v_7 channels with systemic retigabine causes membrane hyperpolarization and suppression of SA in this subpopulation of DRG neurons in STZ-rats. These findings suggest that K_v_7 channels are involved in mechanisms of SA generation in Aβ-LTMs, and taken together with our previous findings of significant attenuation of mechanical hypersensitivity (allodynia) in STZ rats (Djouhri et al., 2019) suggest that retigabine exerts its antiallodynic effects probably by causing membrane hyperpolarization in Aβ-LTMs.

## Data Availability Statement

The datasets generated for this study are available on request to the corresponding author.

## Ethics Statement

The experimental protocols were approved by the University of Liverpool Ethical review committee, and complied throughout with the UK Animals (Scientific Procedures) Act 1986.

## Author Contributions

LD designed the research, conducted some of the electrophysiological experiments, and wrote the first draft of the manuscript. AZ helped in analyzing the behavioral data, writing, editing, and revising the manuscript. TS helped in conducting the behavioral and electrophysiological experiments, writing, editing, and revising the manuscript. SA helped in writing, editing, and revising the MS. All authors approved final version of the manuscript.

## Conflict of Interest

The authors declare that the research was conducted in the absence of any commercial or financial relationships that could be construed as a potential conflict of interest.

## Supplementary Material

FIGURE S1 Changes in AP variables in cutaneous Aβ-LTMs in STZ rats and the effects of retigabine on those variables in Aβ-LTMS with SA. The variables shown are the same as those shown in Figure 3. Note the significant decreases in the CV **(B)** and AHP duration **(H)** and AHP amplitude **(I)**. Note that administration of retigabine (6 mg/kg, i.v.) had not significant effects on all the variables. Details are as in Figure 3. Note that there was no significant changes in the other variables: electrical threshold **(A)**, AP height **(C)**, AP overshoot **(D)**, AP duration **(E)**, AP rise time **(F)**, and AP fall time **(G)**.
