# Supplementary figures and images for: Cutaneous Aβ-Non-nociceptive, but Not C-Nociceptive, Dorsal Root Ganglion Neurons Exhibit Spontaneous Activity in the Streptozotocin Rat Model of Painful Diabetic Neuropathy in vivo
Source: Front Neurosci. 2020 May 25;14:530. doi: 10.3389/fnins.2020.00530 (PMC7263321; doi:10.3389/fnins.2020.00530)

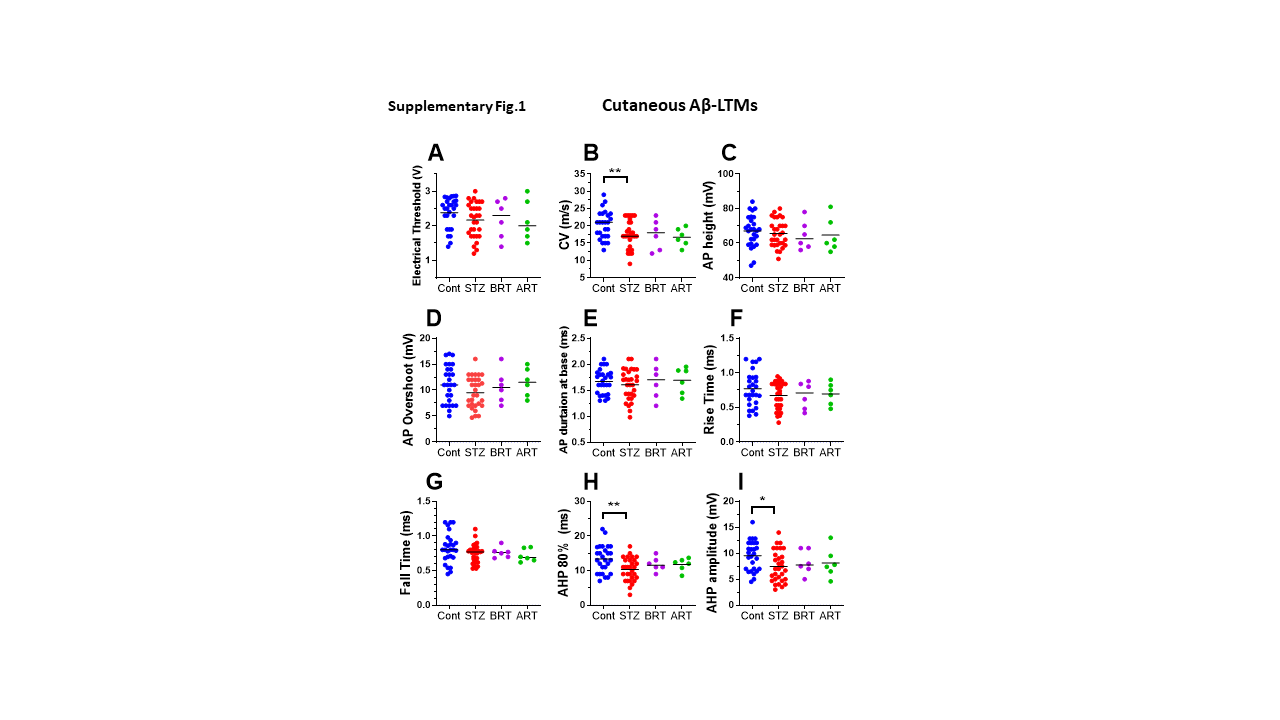

Supplement: FIGURE S1 — Changes in AP variables in cutaneous Aβ-LTMs in STZ rats and the effects of retigabine on those variables in Aβ-LTMS with SA. The variables shown are the same as those shown in Figure 3. Note the significant decreases in the CV (B) and AHP duration (H) and AHP amplitude (I). Note that administration of retigabine (6 mg/kg, i.v.) had not significant effects on all the variables. Details are as in Figure 3. Note that there was no significant changes in the other variables: electrical threshold (A), AP height (C), AP overshoot (D), AP duration (E), AP rise time (F), and AP fall time (G). [file Image_1.TIF]
